# The development and validation of the Social Attributions for Mental Illness (SAMI) scale

**DOI:** 10.1371/journal.pone.0324592

**Published:** 2025-05-23

**Authors:** Leigh Huggard, Finiki Nearchou, Cliódhna O’Connor

**Affiliations:** School of Psychology, University College Dublin, Dublin, Ireland; University of Missouri School of Medicine, UNITED STATES OF AMERICA

## Abstract

Existing measures of lay causal attributions for mental illness do not discriminate between the diverse array of social factors known to influence mental health. Moreover, while ample research has emphasised the negative stigma consequences of biological attributions, limited research investigates how different social attributions might relate to stigma attitudes. The study developed and validated a novel scale to measure lay social attributions for various categories of mental illness. Scale items were generated via data triangulation from an online qualitative survey, a rapid umbrella literature review, and a media analysis. An online survey was disseminated to 500 participants, who rated items’ importance in causing four mental illness categories: anorexia nervosa, depression, schizophrenia, and post-traumatic stress disorder. Exploratory and confirmatory factor analysis identified and validated the factor structure of the Social Attributions for Mental Illness scale (SAMI) for each mental illness. Factors identified were life circumstances, violence/abuse, relational challenges, and sociopolitical turmoil. Tests of validity demonstrated good construct validity. This scale enables investigation of how social attributions may differ across populations and mental illness categories, and the consequences of such differences for attitudes and behaviour.

## Introduction

Public stigma exerts substantial adverse effects on individuals with mental health difficulties [1,2]. An essential avenue for understanding stigma processes involves investigating how members of the public attribute causes to mental illness, which meta-analytic evidence links with stigmatising attitudes [3]. Specifically, while endorsing biological causes of mental illness may reduce blame towards people with mental illness, biological attributions overall cultivate more stigmatising attitudes and therapeutic pessimism [3–5].

While longitudinal evidence suggests biological attributions are growing in popularity [6,7], research indicates many social causes are readily endorsed by laypeople, with psychosocial attributions more prevalent than biological attributions overall [8]. Furthermore, social factors comprise the most common explanations presented in mass media [9], indicating the public are exposed to more social explanations than biological explanations. However, this media research shows the umbrella term ‘social’ encompasses a huge diversity of intrapersonal, interpersonal, environmental, cultural and societal factors [9]. No research enlightens the relative importance laypeople ascribe to these different factors in explaining mental illness. The numerous studies demonstrating the harmful effects of biological attributions commonly use a short, generalised measure of ‘social attributions’, which averages diverse causal factors into a single score, as a control or comparative point [10,11]. This obscures any potential variation in endorsement of different types of social factors (e.g., socioeconomic vs familial factors). This is a notable gap, since different social attributions may have different consequences for attitudes and behaviour. For instance, attributing mental illness to economic inequality could motivate political action, while attribution to unhealthy family dynamics could fuel stigmatisation via ‘social essentialism’ [12]. The idea that different social attributions may have differing relationships to stigma is also supported by both attribution theory [13] and essentialism theory [12,14]: attribution theory predicts that attributions which are controllable and internal should be associated with greater stigma, while essentialism theory predicts that attributions which are deterministic in nature should be linked to greater stigma.

At present, the literature lacks a standardised measure which discriminates between diverse social attributions for mental illness. Existing instruments, such as the Mental Illness Attribution Questionnaire (MIAQ; Knettel, 2019) [15], measure social attributions as a singular construct. More precise differentiation is imperative to establish whether different types of social attributions have distinct attitudinal/behavioural consequences. A differentiated measure would also allow for meaningful comparison of the relative prominence of diverse social attributions across contexts, populations, and mental disorder categories. The latter is particularly important given prior evidence that attitudinal effects of biogenetic attributions differ across mental illnesses [16,17].

### The present study

The study aimed to develop and validate a novel measure; the Social Attributions for Mental Illness (SAMI) scale that assesses lay endorsement of different social factors as determinants of mental illness. Social factors were defined as any aspect of one’s environment, relationships, and life experiences that impacts mental health/illness. For instance, it does not include biological processes, genetics, cognitive factors, behavioural factors, or physical illness. To ensure wide applicability of the scale for research purposes, it was validated according to four categories of mental illness. Scale items were identified via triangulation of three sources of social determinants, reduced and assessed for content validity, and presented to target participants in survey format before being subjected to factor analysis and tested for validity.

## Materials and methods

**Fig 1:**
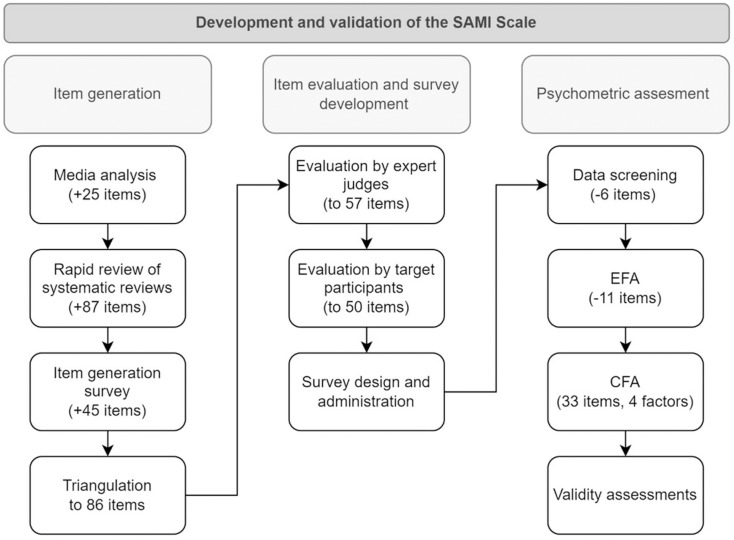
Flowchart for the development and validation of the SAMI.

Following guidelines in best practices in scale development [18,19], development and validation of the SAMI had three phases; *item generation*, *item evaluation and survey development*, and *psychometric assessment* (Fig 1).

### Phase 1: Item generation

A combination of inductive and deductive methods generated a list of items for potential inclusion in the scale. Potential items were procured from three sources and combined using qualitative triangulation via content analysis. The supplementary material (S1 File) shows all items at each iterative stage of the item generation and evaluation process.

#### Media study.

The first source was a previously-conducted media analysis, which examined causal explanations for numerous mental disorders in popular print and online news media in Ireland between March 2018 and March 2022 [9]. The media analysis involved a coding-frame which captured the range of attributions for mental illness offered in news articles. Each social attribution code was extracted from the coding-frame and considered for inclusion.

#### Rapid literature review of systematic reviews.

The second source was a rapid umbrella literature review, which synthesised 37 systematic reviews of the social determinants of mental illness [20]. All evidenced social determinants were considered for inclusion.

#### Item generation survey.

To incorporate direct responses from target-participants, an online questionnaire was disseminated to 120 members of the public (aged 19–72, *M* = 40.1, *SD* = 16.3, 80.8% female) between July and September 2022. The sample size was determined using a data saturation approach to ensure that the list of social factors was sufficiently comprehensive while avoiding unnecessary redundancy [21]. The survey was disseminated via convenience sampling methods, including distributing the survey on social media platforms (Twitter, Reddit, Facebook) and amongst personal contacts, and advertising the survey on a local volunteer centre website (volunteerdlr.i.e.,). The survey contained a single open-ended question which asked participants to list what they believed the non-biological causes of mental illness are. Responses were analysed via content analysis (S2 File shows the coding frame, survey description, and further demographic information). Codes deemed outside the scope of social causes (*n* = 7) were removed. These predominantly represented mental health symptoms (e.g., “depression/pessimism”), or physical factors (e.g., “physical health issues/pain”), as opposed to social factors. All other codes (*n* = 45) were considered for inclusion.

#### Triangulation.

The social factors generated from each of these three sources were combined via data triangulation [22]. Items with low frequency that occurred in either the survey (e.g., “weather and climate change”, *n* = 4, 0.6%) or media analysis (e.g., “lack of parental affection”, *n* = 8, 1.2%) were excluded or merged with other items. While all factors identified in the rapid umbrella review were considered for inclusion, highly specific factors were merged with other items (e.g., “night-time shift work” and “long working hours” were combined to “work/employment factors”). This decision was made as the review contained a wider variety of item types. In addition, while the review took an international perspective, the media study and survey study involved a predominantly Irish/Western European perspective, and consequently certain items (e.g., “natural disasters”), were absent from these despite their potential global relevance.

### Phase 2: Item evaluation and survey development

#### Content validity assessment.

While the comprehensive item generation process ensured items had high content validity, items were further evaluated by target-participants and relevant experts to ensure optimal content validity, clarity and comprehensibility.

##### Expert judges.

Two academic experts within the authors’ professional networks in mental health and causal attribution (FN, CoC) rated the list of preliminary items via an online questionnaire. Experts voted on whether each item should be included in the final scale and made item-specific queries/suggestions. Initial inter-rater agreement was 78.57%. All disagreements were resolved via discussion. Based on the feedback, items underwent further revisions, including re-wording, omitting, and combining items.

##### Evaluation by target participants.

Five members of the public were individually asked to review all items in an informal verbal discussion. Participants were identified using convenience sampling methods (via personal contacts). Each individual was asked to verbalise their mental process of rating the items and identify any unclear or problematic items. All problematic items were amended/omitted as appropriate.

#### Survey design and administration.

The final survey contained 50 items representing diverse social factors. Consistent with prior causal attributions research (e.g., Knettel, 2019; Phelan et al., 2006) [23], four vignettes were developed, each describing DSM-5 (APA, 2013) [24] diagnostic criteria for one of: posttraumatic stress disorder (PTSD), depression, schizophrenia, or anorexia nervosa (S3 File). These psychiatric disorders were chosen to represent mental illnesses whose aetiology involves a diverse range of potential social factors. Public attributions to social factors tend to be more prevalent for PTSD and less prevalent for schizophrenia, with social attributions for depression and anorexia nervosa falling in-between [9]. Inclusion of multiple mental illness prompts ensured the scale’s usability in future research on diverse mental illnesses, and facilitated comparisons of attributions made for different mental illnesses.

Survey data were collected anonymously via an online platform (Pavlovia Surveys) between 24^th^ Feb and 24^th^ March 2023, using the participant recruitment service Prolific (www.prolific.com). Participants consented to the study, were over 18 years, based in Ireland/the UK, and renumerated at Prolific minimum wage rates. The survey was presented in a repeated measures fashion, with each participant sequentially presented with each of the four vignettes, followed by the SAMI and relevant measures for validity assessment (see below). The survey also included demographic questions. The full dataset is available at https://osf.io/4gry9. Ethical approval was granted by University College Dublin’s Research Ethics Committee (approval number: HS-23–03-Huggard-OConnor). Participants provided informed consent by electronically confirming their agreement after reading a study information sheet (S4 File).

#### Participants.

As established guidelines recommend a sample size of approximately 250 participants for both EFA and CFA to ensure adequate power and reliable factor structure [18,25], a total of 500 participants were recruited to the survey. Table 1 shows participant demographic information.

**Table 1:** Demographic information for the total sample (*n* = 500).

Age	Range	18 - 82
	*M*	38.85
	*SD*	13.89
Gender	Male	239 (48.7%)
	Female	249 (50.7%)
	Other	3 (.6%)
Political affiliation	Left-wing	228
	Centre	202
	Right-wing	66
Nationality	UK	390 (86.5%)
	Ireland	21 (4.7%)
	Other	40 (8.9%)
Ethnicity	White Irish/British	408 (82.6%)
	White other	33 (6.7%)
	Black Irish/British	5 (1.0%)
	Black other	4 (.8%)
	Asian Irish/British	9 (1.8%)
	Asian other	19 (3.8%)
	Other/Mixed	16 (3.2%)
Education	Primary education	5 (1.0%)
	Junior secondary education	7 (1.4%)
	Higher secondary education	110 (22.2%)
	Third level education	278 (56.0%)
	Postgraduate education	96 (19.4%)
Understanding of depression	Had never heard of it	1 (.3%)
	Knew a little	209 (54.9%)
	Knew a lot	150 (39.4%)
	Expert	21 (5.5%)
Understanding of schizophrenia	Had never heard of it	3 (.6%)
	Knew a little	428 (87.0%)
	Knew a lot	57 (11.5%)
	Expert	4 (.8%)
Understanding of PTSD	Had never heard of it	2 (.4%)
	Knew a little	364 (74.1%)
	Knew a lot	118 (24.0%)
	Expert	7 (1.4%)
Understanding of anorexia nervosa	Had never heard of it	12 (2.4%)
	Knew a little	377 (76.0%)
	Knew a lot	100 (20.2%)
	Expert	7 (1.4%)
Knows someone with	Depression	364 (73.0%)
	PTSD	74 (14.8%)
	Anorexia nervosa	131 (26.3%)
	Schizophrenia	105 (21.0%)
	None	108 (21.6%)
If yes, was this person	An acquaintance	127
	A close friend	145
	A family member	186
	Yourself	140

#### Measures.

##### Social attributions for mental illness (SAMI) scale:

For each mental illness condition, the full list of potential items (*n* = 50) was presented to participants using the following prompt: “*We would like to know how important you believe each factor is in causing [depression/schizophrenia/PTSD/anorexia nervosa]. Please rate each of the following items on a scale from 1 to 5, where 1 indicates that factor is “not at all important” and 5 indicates it is “extremely important” as a cause of [depression/schizophrenia/PTSD/anorexia nervosa]”.* A range of 1–5 was chosen based on recommendations for best practices in scale development [18]. SAMI items were presented in randomised order. Scale scores for each subscale were calculated by averaging item scores, with higher scores indicating greater endorsement of the attribution as a cause of mental illness. For further instructions regarding use of the SAMI, see the supplementary material (S5 File).

##### Mental illness attribution questionnaire (MIAQ), social/stress subscale and heredity/biological subscale

[15]. General attributions to social/stress factors (e.g., “traumatic experience”) for each mental illness was used to test for convergent validity. We expected the MIAQ social/stress subscale would be a strong, positive predictor of SAMI subscales. Attributions to heredity/biological factors (e.g., “accidental brain injury”) was used to test for divergent validity. Based on prior research [5,15] we expected the heredity/biological subscale to be a weak to moderate predictor of SAMI subscales. MIAQ items were presented with the following prompt “*Please continue to rate each of the following items on a scale from 1 to 5, where 1 indicates the factor is “not at all important” and 5 indicates the factor is “extremely important” as a cause of [depression/schizophrenia/PTSD/anorexia nervosa].* In the present sample, internal consistency ranged between α = 0.84 and 0.93 across mental illness conditions for the heredity/biological subscale, and α = 0.93 and 0.97 across conditions for the social/stress subscale.

##### Political affiliation

We used a single item self-report measure to test for discriminant validity. Each participant rated their political affiliation on a five-point scale from 1 = very left-wing to 5 = very right-wing. We expected that participants identifying as left-wing would report significantly higher attributions to social factors than participants identifying as right-wing [26].

### Phase 3: Psychometric assessment

#### Data analysis overview.

The sample was randomly split into two sub-samples for the analysis. Data from Sample 1 (*n = *250) was used to estimate the factor structure via exploratory factor analysis (EFA). Data from Sample 2 (*n = *250) was used to establish the factor structure via confirmatory factor analysis (CFA) and to assess reliability and validity. The supplementary material contains demographic characteristics broken down by sample (S6 File). As the study was presented in repeated measures format, each sample contained four sets of responses per participant, one for depression, anorexia nervosa, schizophrenia, and PTSD.

To establish data’s suitability for factor analysis, the Kaiser-Meyer-Olkin (KMO) measure of sampling adequacy (values >0.8) and Bartlett’s test of sphericity (*p* < 0.05) were utilised, with KMO values greater than.8 and significant scores on Bartlett’s test (*p* < 0.05) indicating factorability.

EFA using principal axis factoring was used to assess the structure of the SAMI. To establish the optimal factor structure for all four mental illness types, five EFAs were conducted – one for the combined (i.e., all four conditions) Sample 1 data, and one for each of the four Sample 1 conditions individually. The number of factors to retain was determined based on (1) Kaiser’s Rule, where factors with eigenvalues >1 were retained, (2) visual identification of scree plots and (3) the number of factors that collectively explained over 50% of the variance in the data. As the factors were hypothesised to correlate, oblique rotation (Promax) was used.

CFA was applied to the Sample 2 data to confirm whether the resulting hypothesised factor structure fit the data adequately. To establish whether the measurement model was consistent across each of the four conditions, the Sample 2 data was subjected to invariance testing [27]. Invariance testing is a common statistical procedure used to assess whether the measurement properties of a latent construct remain stable across multiple waves of data collection (e.g., 24,25). This involves sequentially evaluating the fit of (1) configural invariance to confirm the presence of the same latent factors within each condition, (2) metric (weak) invariance to ensure consistent relationships between latent variables and observed item variables, and (3) scalar (strong) invariance to verify intercept invariance and measurement consistency. The estimator used was weighted least square mean and variance (WLSMV), as it provides less biased estimates for ordinal data than maximum likelihood with robust standard errors (Li, 2016). Fitting adequacy was established by using each of the following indices: Tucker Lewis Index (TLI) and Comparative Fit Index (CFI) with values >0.90 indicating good fit and values ≥0.95 indicating excellent fit; Root Mean Square Error of Approximation (RMSEA), and Standardized Root Mean Square Residual (SRMR) with values ≤0.08 indicating good fit. The chi-square test of exact fit (*χ*^2^) was not utilized to establish fit in the present analysis as it is sensitive to sample size and likely to provide biased estimates [25].

The internal consistency of each resulting construct was assessed via Cronbach’s alpha reliability coefficients. Based on recommendations for validity testing [18], a series of simple linear regression analyses were conducted to test for convergent and divergent validity, with SAMI subscales as dependent variables, and MIAQ subscales as independent variables. Discriminant validity was assessed via a series of independent samples *t*-tests, with SAMI scores as dependent variables and political affiliation (right- and left-wing) as the independent variable [28,29].

All analyses were conducted using RStudio version 2023.6.1.524. Packages used included *lavaan* (v0.6-15; Rosseel, 2023) and *psych* (v2.3.6; Revelle, 2023). The study was preregistered on Open Science Framework (OSF) prior to data collection (https://doi.org/10.17605/OSF.IO/KSCR8).

## Results

### Data screening and missing data

Sample 1 had 1.35% missing data, and Sample 2 had 1.17% missing data. As Little’s MCAR test indicated data was not missing completely at random, imputation using expectation maximisation (EM) was conducted on the data. Tests of skewness and kurtosis revealed the data was approximately normally distributed.

### Exploratory factor analysis

The Sample 1 data supported factorability and was deemed suitable for EFA: the correlation matrix indicated all but one of the correlation coefficients were greater than 0.3, the KMO value was 0.98, and Bartlett’s Test of Sphericity was significant, $\chi^{\text{2}}\left(\text{1225}\right)\text{=47584.97, p}<.\text{001}$. Four items were removed prior to factor analysis due to very high correlations (*r* > 0.8) with other items (“job insecurity”, “unemployment”, “insufficient or insecure income”, “poor working conditions”), one item was removed due to low correlations (*r* < 0.3) with other items (“beauty/fitness standards”), and one item was removed due to a high degree of negative correlation with other items (“social media”). Based on a combination of the scree plot, eigenvalues, and the percentage of variance accounted for by the factors from each condition, a four-factor solution was deemed optimal.

The pattern matrices of each of the five EFAs were evaluated to assess salience and simple structure. A cut off point of 0.4 was used [30]. Items were removed due to weak loadings (*n* = 2), lack of theoretical association with other items in the construct (*n = *2), and high cross-loadings (*n* = 7), denoted either by multiple items greater than.4, or items with differences in cross-loadings less than 0.2 [30]. The remaining items and their cross loadings from the Sample 1 data combining all mental illness conditions are presented in Table 2. The four constructs were labelled as follows: *Life Circumstances*, *Violence/Abuse*, *Relational Challenges*, *and Socio-Political Turmoil*.

**Table 2:** Factor structure of the SAMI.

Items	*Life Circumstances*	*Violence/* *Abuse*	*Relational Challenges*	*Socio-Political Turmoil*
Reproductive struggles/ infertility	0.97			
Poor housing conditions	0.78			
Social isolation/loneliness	0.77			
Insufficient leisure time, lack of time off work	0.74			
Inadequate social welfare supports	0.72			
Poverty	0.70			
Income inequality	0.69			
Lack of access to healthcare	0.69			
Lack of social support	0.68			
Unstable living conditions, moving around too often	0.66			
Caregiving burden	0.64			
Stressful job	0.57			
Being displaced from one’s home	0.56			
Child sexual abuse		0.95		
Child physical abuse		0.89		
Child emotional abuse		0.88		
Child neglect		0.75		
Domestic violence		0.71		
Physical abuse in adulthood		0.71		
Sexual harassment		0.51		
Events that cause physical harm/injury		0.45		
Negative influences from social groups			0.97	
Not fitting in			0.87	
Gender norms			0.77	
Relationship issues (with a romantic partner)			0.66	
Pressure and expectations			0.60	
Contact with other people with mental illness (in person/online)			0.50	
Protests, riots, and revolutions				0.95
Natural disasters				0.93
Armed conflict				0.79
Violence within the neighbourhood				0.75
Political instability				0.67
Stress related to migration				0.58
Eigenvalues	8.24	5.88	5.37	5.75
Variance explained per factor	18.7%	13.4%	12.2%	13.1%
Cumulative variance	18.7%	32.1%	44.3%	57.4%

Communalities are based on data from the combined EFA using Sample 1 data. Only values > .4 are presented.

### Confirmatory factor analysis

To validate the factor structure of the SAMI, CFA analyses were applied to the Sample 2 data. However, as the sample size within each Sample 2 condition (*n* = 250) produced insufficient power for the analysis and resulted in inflated fit indices, Sample 2 data was combined with Sample 1 data for the purposes of each CFA analysis.

To establish whether the hypothesised model remained consistent across conditions, invariance testing was applied to the data. The results of the configural invariance test demonstrated a satisfactory fit, indicating the proposed measurement model was consistent across each of the four conditions (see Table 3 for fit statistics). Analysis of metric invariance, however, yielded a significantly poorer fit compared to the configural model $\chi^{2}(87)=516.1,\;p<\mathrm{.001}$, suggesting the strength of the relationship between the observed items and the latent factors varied across conditions. Upon inspection of the item parameters, approximately 66% of the items in the model varied across at least two conditions, with fewer varying across multiple conditions. Despite these slight variations in the relationships between some of the observed indicators and the underlying latent construct, such differences were theoretically expected given that each of the mental illness vignettes were designed to prompt differing responses, and some items should predictably hold different meaning across different mental illnesses. As the underlying constructs were deemed conceptually coherent, and a substantial proportion of items were invariant across conditions, this justifies the use of the model in future research to meaningfully compare responses across conditions/mental illness types.

**Table 3:** CFA fit statistics.

Model	CFI	TFI	RMSEA	SRMR
Configural model	0.989	0.988	0.022	0.207
Metric model	0.949	0.947	0.046	0.245
Scalar model	0.935	0.935	0.051	0.234
Anorexia nervosa	0.988	0.987	0.051	0.072
Depression	0.995	0.995	0.024	0.054
PTSD	0.976	0.974	0.062	0.083
Schizophrenia	0.999	0.999	0.006	0.043

Because the data demonstrated partial invariance, the decision was made to conduct separate CFAs for each of the four conditions (as opposed to a single CFA with the combined data) to ensure the model demonstrated good fit for each mental illness condition. All models displayed satisfactory fit to the data (see Table 3), supporting the validity of the measurement model in each condition.

### Reliability

Cronbach’s alpha coefficients for each of the SAMI subscales using Sample 2 data indicated excellent internal consistency across each mental illness condition (Table 4). No items were removed following reliability assessments, as the analysis predicted no substantial improvements in internal consistency were any item removed from any subscale.

**Table 4:** Internal consistency measured via Cronbach’s alpha for each of the SAMI subscales using Sample 2 data.

SAMI subscale	Cronbach’s alpha
(1) Life circumstances	0.96
(2) Violence/abuse	0.95
(3) Relational challenges	0.89
(4) Sociopolitical turmoil	0.92

### Validity assessments

Validity assessments were conducted using Sample 2 data (see S7 File for validity results).

Simple linear regression analyses were conducted for each mental illness condition to assess whether each subscale of the SAMI was a significant predictor of MIAQ social/stress. All analyses were statistically significant (*p* < 0.05), with the predictor explaining a large proportion of the variance in each analysis (ranging from 22% to 83%), demonstrating that the SAMI presents good convergent validity.

Linear regression analyses were also conducted between each SAMI subscale and the MIAQ heredity/biological subscale. Consistent with previous research [5,15], each SAMI subscale either weakly or moderately predicted the MIAQ heredity/biological subscale, indicating that the SAMI presents adequate divergent validity, i.e., SAMI attributions are conceptually distinct from heredity/biological attributions.

Discriminant validity was assessed using self-rated political affiliation, where SAMI scores were expected to be higher for participants identifying as having left-wing than right-wing political affiliations. For each condition, independent samples *t*-tests revealed a significant difference in SAMI subscale scores between left- and right-wing participants, with the exception of three; irrespective of political affiliation, participants reported similar attributions of PTSD to *relational challenges* and *life circumstances*, and similar attributions of anorexia to *sociopolitical turmoil*. Despite the three tests indicating no significant differences, left-wing participants demonstrated higher social attributions when compared to right-wing participants in the 13 other tests, demonstrating that the SAMI shows good discriminant validity overall.

## Discussion

This paper presents the development and validation of the SAMI: a novel tool designed to measure four types of social attributions for mental illness; attributions to life circumstances, violence/abuse, relational challenges, and sociopolitical turmoil.

Items were developed via qualitative triangulation of social factors derived from three sources, assessed for content validity, reduced, and presented to participants in survey format. Exploratory factor analysis identified a four-factor structure of the SAMI, which was validated by confirmatory factor analysis.

Analysis of configural invariance indicated that the structure of the measurement model remained consistent across all four mental illness conditions. Despite the metric model displaying worse fit than the configural model, the scale retains its utility. The use of vignette conditions is a widely adopted method in attribution research (e.g., Angermeyer & Matschinger, 2005; Knettel, 2019; Phelan et al., 2006) [15,23,31], and partial invariance recognises that not all items need to be invariant for meaningful comparisons [32]. Moreover, given the intentional design of the vignettes to prompt varying responses, it is theoretically expected that the relationship between items and latent variables might differ. For instance, the item “child neglect” as a cause for PTSD may be understood as childhood experiences of chronic stress or traumatic events, whereas for depression, may be understood as a lack of emotional support or nurturing. While slightly different, both interpretations are pertinent to the construct of *violence/abuse* and retain their validity*.* As such, the SAMI is a valid and valuable tool for comparing endorsement of attributions across mental illnesses.

The analysis also indicated good internal reliability, and strong evidence of construct validity, with each SAMI construct displaying convergent validity by strongly predicting MIAQ social/stress scores, and divergent validity by weakly/moderately predicting MIAQ heredity/biological scores. Additionally, the majority of SAMI scores differed among political affiliation, exhibiting discriminant validity.

### Strengths and limitations

The study was designed according to best practice in scale development [18,19]. The intensive and systematic item generation process ensured a high degree of content and face validity within the items, ensuring a wide range of ecologically valid items were included in the scale. However, while the rapid umbrella review took an international perspective, the media study was based in Ireland only, and the item survey and scale survey included participants predominantly from Ireland and the UK. We therefore recommend that future research validate the scale for use in other cultural contexts, particularly non-western cultures where attributions are likely to differ [8,33]. Moreover, while the use of an online recruitment platform (Prolific) for the main survey offers advantages over traditional convenience samples, such as greater participant attentiveness and broader demographic diversity [34], this method of recruitment may have introduced potential sample limitations, such as underrepresentation of hard-to-reach samples and self-selection bias [35].

Another potential limitation relates to the absence of evidence regarding the SAMI’s predictive and concurrent validity. Since, to our knowledge, no prior attribution research has distinguished between specific social attributions, we were unable to make informed predictive hypotheses. We were similarly unable to assess whether the SAMI presents concurrent validity without a “gold standard” construct of this nature.

Additionally, while best practice guidelines recommend involving approximately 5–7 expert judges, and 5–15 target participants over 2–3 rounds for the Phase 2 content validity assessment, only two expert judges and five target participants were recruited. This decision was made due to limited resources and because all reviewers consistently identified similar items as unclear/problematic, suggesting that further reviews may have resulted in unnecessary redundancy and participant burden.

Lastly, while the high internal consistency (α > 0.90) of some subscales reflects strong item coherence, it may also indicate potential item redundancy, raising the possibility that some subscales, particularly the *life circumstances* and *violence/abuse* subscales, could be shortened. However, the high alpha values may be explained by the overlap of participants across EFA and CFA analysis samples. While this overlap ensured adequate statistical power and more reliable fit statistics for the CFA analyses, it may have led to artificially high alpha values [36].

### Implications

The SAMI represents a promising tool with various avenues for use in future research. Firstly, it offers a valuable resource for mental health literacy research, enabling deeper exploration of the public’s attributions of mental illness to specific social factors. Existing research has revealed the public tend to underestimate the role of social determinants in physical illness [37]; the SAMI could help determine if analogous patterns exist for mental illnesses.

Secondly, the SAMI holds substantial potential for mental health stigma research. Previous studies have indicated that, overall, social attributions for mental illness are associated with less stigmatising attitudes than biological attributions [3–5]. The SAMI adds further nuance by identifying the particular social attributions that predict the most inclusive/tolerant attitudes – an approach that has already been adopted in recent research [38]. This insight may have far-reaching implications for the development of anti-stigma interventions.

Moreover, while the SAMI was designed for use in the general population, it offers opportunities for validation in alternative populations, including clinical populations and mental health practitioners. As practitioners’ perceptions of mental illness determinants can significantly impact treatment outcomes [11,39], extending the SAMI’s application to these groups may yield important insights.

## Conclusions

The SAMI is a viable tool for measuring social attributions for mental illness. The four constructs identified cover various aspects of the social world, from malleable lifestyle and situational factors, to interpersonal relationships, to wider environmental and political contexts. This instrument will further understanding of the relationships between causal attributions and stigma attitudes in the general population. The outcomes of this improved understanding promise great value for stigma reduction efforts, identifying the specific attributions to encourage or counteract in efforts to promote the social inclusion of people with mental health difficulties.

## Supporting information

S1 File Items at each iterative stage of item generation and development. (DOCX)

S2 File Item generation survey information. (DOCX)

S3 File Survey vignettes. (DOCX)

S4 File Participant information sheet. (DOCX)

S5 File Instructions for use of the SAMI. (DOCX)

S6 File Demographics by sample. (DOCX)

S7 File Validity and reliability analysis results. (DOCX)
