# Supplementary material for: The development and validation of the Social Attributions for Mental Illness (SAMI) scale
Source: PLoS One. 2025 May 23;20(5):e0324592. doi: 10.1371/journal.pone.0324592 (PMC12101631; doi:10.1371/journal.pone.0324592)
Supplement: S1 File — (DOCX) [file pone.0324592.s001.docx]

# List of items identified from the media analysis (Huggard & O’Connor, 2023)

Cultural, societal environment - OTHER

Cultural, societal expectations

Overexposure to fear-inducing or distressing information

Social media, the internet

Family environment - OTHER

Having a parent with physical or mental health issues

Lack of parental attention or affection

Educational pressures

Environmental factors (unspecified)

Grief, losing a loved one

Life events, experiences - OTHER

Pregnancy, childbirth

Stressful experiences

Trauma, negative experiences and abuse

Emotional self-management

Personality, disposition, character

Psychological factors - OTHER

Contact with other people with mental illness

Mistreatment by others

Social isolation, loneliness, COVID-19 related lockdowns

Social, interpersonal relations - OTHER

Employment conditions

Financial strain, poverty

Housing and living conditions

Socio-economic conditions - OTHER

# List of items identified from the rapid literature review of systematic reviews (Huggard et al., 2023)

Domestic violence

Childhood or school violence

Bullying

Armed conflict

Verbal abuse

Sexual abuse

Neighbourhood crime

Child abuse and neglect

Child marriage

Protests, riots and revolutions

Natural disasters

Events that cause physical harm/injury

Childbirth

Reproductive events

Death of family members

Caregiving burden

Childhood adversity

Parent-child relationships

Dysfunctional family life

Racism

Discrimination

Being displaced from ones home

Factors related to migration

Perceived social inequality

Low social support

Difficulties with interpersonal relationships

Acculturative stress

Social cohesion

Social capital

Culture

Social class

Social and community context

Lack of social support

Social isolation

Quarantine

Reductions in human mobility

Colonialism

Social policy

Child welfare

Residential schools

Historical/intergenerational trauma association with colonialism

Lack of comprehensive and gender inclusive welfare states

Austerity measures

Welfare state regimes

Income inequality

Income insecurity

Debt/assets

Food insecurity

Macroeconomic recessions

Financial strain

Lower household income

Large financial loss

Job insecurity

Precarious employment

Occupations such a rescue worker or ambulance worker during disasters/wars, healthcare worker or family member of a healthcare worker during the COVID-19 pandemic

Retirement

Working conditions

Night-time shift work

Job strain

Psychosocial job stressors

Unemployment

Long working hours

infrastructure

Aggregated socioeconomic deprivation

Leisure opportunities

Urbanicity

Crime

Community violence

Social cohesion

The built environment

Living alone

Living with family

Insecure tenure and mobility

Housing conditions

Discrimination in accessing housing

Overcrowding

Low sense of safety at home

Social connections in neighbourhood

Being female

Being male

Older age

Younger age

Being married

Being single

Ethnicity

High education status

Low education status

# List of items identified from the qualitative item generation survey

Bullying, discrimination and maltreatment

Abuse

lack of support/guidance

Loneliness, isolation and not fitting in (including COVID isolation)

Friends and social environment

death/grief/bereavement

Contracting symptoms from other people/news/online

relationship issues (romantic partner)

Poverty and financial issues

Living conditions

Work/employment factors

work/life balance, lack of days off work

exam/educational pressures

stress/anxiety/worry

pressure/expectations/burnout

depression/pessimism/life dissatisfaction

poor self care, self-regulation, and insight

personal values

Low self-esteem/self-doubt

personality/attitude

health issues/illness including pain

addiction and substance use (including gambling)

sleep/exhaustion/rest

lack of exercise

diet

childhood factors

family environment/parental factors

reproductive struggles or pregnancy

experiences/life changes

trauma

circumstances/environment

weather and climate change

war

culture

religion/spiritual practices

societal norms, traditions, and culture

political climate

cultural cohesion/heterogeneity

resource access/treatment access

social media and technology

disturbing or manipulative online/news content

minority/marginalised people

social class

single

ethnicity

# List of items following the triangulation procedure

domestic violence

armed conflict

verbal abuse

physical abuse

bullying

rape

sexual harassment

violence within the neighbourhood

public shootings

protests, riots, and revolutions

child sexual abuse

child physical abuse

child emotional abuse

child neglect

natural disasters

events that cause physical harm/injury

childbirth

pregnancy

reproductive events/struggles

grief/losing a loved one

caregiving burden

childhood adversity

parent-child relationships

dysfunctional family life

change in life circumstances

discrimination due to race

discrimination due to ethnicity

discrimination due to sex

discrimination due to gender identity

discrimination due to religion

belonging to a minority/marginalised group

lack of comprehensive and gender inclusive welfare states

being displaced from one's home

perceived social inequality due to migration

difficulties with interpersonal relationships due to migration

stress related to moving into a new culture

lack of a bond with people in the culture you live in

pressure, expectations and burnout

gender norms

beauty standards

fitness standards and athletic expectations

pressure to eat well

fame, being in the public eye

overexposure to fear-inducing or distressing information

disturbing or manipulative online/news content

social media, the internet and technology

lack of social support

social isolation

contact with other people with mental illness (in person or online)

loneliness

not fitting in

negative influences from social environment /friend group

relationship issues (with a romantic partner)

political climate

welfare state regimes

policy decisions

lack of access to healthcare

lack of access to education

lack of access to childcare

lack of access to a diagnosis

food insecurity

austerity measures

income inequality

having insecure or unstable income

being in debt

macroeconomic recessions

financial strain

lower household income

poverty

job insecurity

having a stressful occupation

precarious employment

retirement

poor working conditions

unemployment

pressures related to education

work-life balance / lack of days off work

lack of opportunities for leisure in one's neighbourhood

living in an urban/city environment

not feeling connected to other members of the neighbourhood

poor housing conditions

living alone

living with family

unstable living conditions

overcrowding

lack of social connections in one's neighbourhood

# List of items following evaluation by experts

domestic violence

armed conflict

verbal abuse

physical abuse in adulthood

bullying

sexual assault and rape

sexual harassment

violence within the neighbourhood

protests, riots, and revolutions

child sexual abuse

child physical abuse

child emotional abuse

child neglect

natural disasters

events that cause physical harm/injury

pregnancy and birth

reproductive struggles, infertility

grief/losing a loved one

caregiving burden

dysfunctional family dynamics

discrimination due to race/ethnicity

discrimination due to sex/gender identity

discrimination due to religion

being displaced from one's home

stress related to migration

pressure, expectations and burnout

gender norms

beauty/fitness standards

overexposure to fear-inducing or distressing information

social media

lack of social support

social isolation/loneliness

contact with other people with mental illness (in person or online)

not fitting in

negative influences from social groups

relationship issues (with a romantic partner)

political instability

inadequate social welfare supports

lack of access to healthcare

lack of access to education

food insecurity

financial austerity measures

income inequality

debt

insufficient or insecure income

poverty

job insecurity

stressful job

retirement

poor working conditions

unemployment

educational pressures

insufficient leisure time, lack of time off work

poor housing conditions

living alone

unstable living conditions, moving around too often

overcrowding

# List of items following evaluation by target participants

Being displaced from one's home

Caregiving burden

Inadequate social welfare supports

Income inequality

Insufficient leisure time, lack of time off work

Lack of access to healthcare

Lack of social support

Poor housing conditions

Poverty

Reproductive struggles/ infertility

Social isolation/loneliness

Stressful job

Unstable living conditions, moving around too often

Child emotional abuse

Child neglect

Child physical abuse

Child sexual abuse

Domestic violence

Physical abuse in adulthood

Sexual harassment

Gender norms

Negative influences from social groups

Not fitting in

Pressure and expectations

Relationship issues (with a romantic partner)

Contact with other people with mental illness (in person/online)

Armed conflict

Natural disasters

Political instability

Protests, riots, and revolutions

Stress related to migration

Violence within the neighbourhood

Dysfunctional family dynamics

Overexposure to fear-inducing or distressing information

Rape and serious sexual assault

Discrimination due to religion

Bullying

Discrimination due to race/ethnicity

Educational pressures

Events that cause physical harm/injury

Food insecurity

Grief/losing a loved one

Verbal abuse

Challenging experience of pregnancy/childbirth

Job insecurity

Unemployment

Insufficient or insecure income

Poor working conditions

Beauty/fitness standards

Social media
