# Supplementary material for: The development and validation of the Social Attributions for Mental Illness (SAMI) scale
Source: PLoS One. 2025 May 23;20(5):e0324592. doi: 10.1371/journal.pone.0324592 (PMC12101631; doi:10.1371/journal.pone.0324592)
Supplement: S2 File — (DOCX) [file pone.0324592.s002.docx]

**Item generation survey study information**

Participants were presented with the following survey prompt:

*Aside from biological factors like genetics, what do you think can cause mental illness?*

*Please list factors that you believe either:*

*1. Can cause mental illness or poor mental health*

*2. Can make mental illness or mental health worse*

*PLEASE NOTE:*

*• DO NOT list biological causes of mental illness such as genetics, brain injury, chemical imbalance in the brain, etc.*

*• DO list any other causes of mental illness*

*• Causes can be for any type of mental illness, such as anxiety, depression, bipolar, schizophrenia, PTSD, etc. You may be as specific or general as you would like.*

**Participants**

Participants were 120 members of the public aged between 19 and 72 (*M* = 40.1, *SD* = 16.3), identifying as female (*n =* 85, 80.8%), male (*n =* 33) and non-binary/third gender (*n =* 2). Most participants had completed third level education or higher (*n =* 99, 82%), and almost all completed at least higher secondary level education (*n =* 119, 99%). Participants were White Irish (*n =* 79, 65.8%), any other White background (*n =* 23, 19.2%), any other Asian background (*n =* 8, 6.7%), any other Black background (*n =* 2, 1.7%), and other/Mixed background (*n =* 8, 6.7%).

**Coding Frame**

Table 1: Codes and frequencies identified through content analysis of the item generation survey results

| **Code** | **Frequency** |
| --- | --- |
| Stress/anxiety/worry | 45 |
| Trauma | 39 |
| Poverty and financial issues | 38 |
| Loneliness, isolation and not fitting in (including COVID isolation *n* = 2) | 37 |
| Abuse | 34 |
| Addiction and substance use (including gambling) | 33 |
| Bullying, discrimination, and maltreatment | 28 |
| Work/employment factors | 28 |
| Family environment/parental factors | 28 |
| Death/grief/bereavement | 27 |
| Childhood factors | 26 |
| Health issues/illness including pain | 21 |
| Experiences/life changes | 18 |
| Social media and technology | 18 |
| Friends and social environment | 17 |
| Living conditions | 16 |
| Diet | 14 |
| Lack of support/guidance | 12 |
| Poor self care, self-regulation and insight | 12 |
| Low self-esteem/self-doubt | 12 |
| Pressure/expectations/burnout | 11 |
| Sleep/exhaustion/rest | 10 |
| Exam/educational pressures | 8 |
| Personality/attitude | 8 |
| Resource access/treatment access | 8 |
| Relationship issues (romantic partner) | 7 |
| Depression/pessimism/life dissatisfaction | 7 |
| Lack of exercise | 7 |
| Circumstances/environment | 6 |
| War | 5 |
| Work/life balance, lack of days off work | 4 |
| Reproductive struggles or pregnancy | 4 |
| Weather and climate change | 4 |
| Disturbing or manipulative online/news content | 4 |
| Societal norms, traditions, and culture | 4 |
| Religion/spiritual practices | 3 |
| Minority/marginalised people | 3 |
| Personal values | 2 |
| Political climate | 2 |
| Cultural cohesion/heterogeneity | 2 |
| Social class | 2 |
| Contracting symptoms from other people/news/online | 1 |
| Sub-cultures e.g., cults/conspiracy groups | 1 |
| Being single | 1 |
| Being of certain ethnicity | 1 |
