# Supplementary material for: The development and validation of the Social Attributions for Mental Illness (SAMI) scale
Source: PLoS One. 2025 May 23;20(5):e0324592. doi: 10.1371/journal.pone.0324592 (PMC12101631; doi:10.1371/journal.pone.0324592)
Supplement: S3 File — (DOCX) [file pone.0324592.s003.docx]

**Survey Vignettes**

**Vignette 1:**

Depression is a type of mental illness. Common symptoms amongst people with depression include low mood, weight changes, difficulty sleeping, feelings of restlessness, fatigue, difficulty concentrating, and suicidal thoughts.

Below is a list of possible causes for depression.

Please rate each of the following items from “not at all important” to “very important” as causes for depression.

**Vignette 2:**

Schizophrenia is a type of mental illness. Common symptoms amongst people with schizophrenia include delusions, hallucinations, disorganised speech or behaviour, or losing the ability to do things such as going to work or school.

Below is a list of possible causes for schizophrenia.

Please rate each of the following items from “not at all important” to “very important” as causes for schizophrenia.

**Vignette 3:**

Anorexia nervosa is a type of mental illness. Common symptoms amongst people with anorexia nervosa include restricting food intake, severe weight loss, intense fear of gaining weight, and body image disturbance.

Below is a list of possible causes for anorexia nervosa.

Please rate each of the following items from “not at all important” to “very important” as causes for anorexia nervosa.

**Vignette 4:**

PTSD (posttraumatic stress disorder) is a type of mental illness. Common symptoms amongst people with PTSD include recurrent and intrusive distressing memories, recurrent distressing dreams, flashbacks, and psychological distress following specific triggers.

Below is a list of possible causes for PTSD.

Please rate each of the following items from “not at all important” to “very important” as causes for PTSD.

**Item scale**

1 = not at all important as a cause, 2 = not very important, 3 = somewhat important, 4 = important, 5 = very important as a cause
