# Supplementary material for: The development and validation of the Social Attributions for Mental Illness (SAMI) scale
Source: PLoS One. 2025 May 23;20(5):e0324592. doi: 10.1371/journal.pone.0324592 (PMC12101631; doi:10.1371/journal.pone.0324592)
Supplement: S4 File — (DOCX) [file pone.0324592.s004.docx]

**Public beliefs about the causes of mental illness**

**Researchers:** Ms Leigh Huggard, Dr Cliodhna O’Connor, Dr Finiki Nearchou

Before you decide if you would like to take part in this research survey, you need to understand why the research is being done and what it would involve for you. Participants will be paid a fee of £2 for participation. Please take time to read the following information carefully.

**What is this research about, and why is it being done?** The purpose of this study is to gain a better understanding of what people believe causes different types of mental illness. The data collected from this study will be used to develop and validate a scale measure which assesses public attitudes around the causes of mental illness.

**Why have I been invited?** Anyone over the age of 18 is welcome to participate.

**How will my data be used?** Your data will be completely anonymous. The data will be archived indefinitely and made publicly available to other researchers. The study findings may be presented and/or published in academic journals and at conferences.

**What will happen if I decide to take part in this research study?** You will be asked to read a short piece of text describing what depression/PTSD/anorexia nervosa/schizophrenia is, followed by a question asking you to rate, on a five-point scale, the extent to which you believe different factors could cause this mental illness. This will be then repeated for each of the remaining three descriptions of a mental illness. You will also be presented with some other questions including demographic questions.

**How will my privacy be protected?** All data collected will be completely anonymous, and your data will be given a research code.

**What are the benefits of taking part?** We cannot promise the study will help you but the information we get from the study will help to increase understanding of public attitudes surrounding mental illness.

**What are the risks of taking part?** As the topic of this survey is on the possible causes of mental illness, there is a chance that the contents of this survey could trigger distress or discomfort in some participants. For example, brief reference will be made to traumatic experiences such as child abuse and sexual violence. If exposure to such concepts might cause you distress, it is best not to participate. If you do participate and at any point you decide to leave the study, contact information for listening services will be presented. These will also be presented at the end of the study.

**Can I change my mind at any time and withdraw from the study?** Participants are free to leave the study at any time without repercussions. However, as all data are collected anonymously, we can’t guarantee that we will be able to delete your data if you have already completed the study.

**Who is organising the research, and how can I find out what happens next with the project?** This research is being completed as part of a psychology research PhD at University College Dublin. If you wish to be informed in the event that this study is published, please contact the researcher at [leigh.huggard@ucd.connect.ie](mailto:leigh.huggard@ucd.connect.ie)

**What if there is a problem?** If you have a concern about any aspect of this study, you should ask to speak to the researcher or research supervisor who will do their best to answer your questions.
Researcher: Leigh Huggard, [leigh.huggard@ucdconnect.ie](mailto:leigh.huggard@ucdconnect.ie)
Research supervisor: Dr Cliodhna O’Connor, [cliodhna.oconnor1@ucd.ie](mailto:cliodhna.oconnor1@ucd.ie)
These contact details will be presented again at the end of the study.

If at any point you find the content of this study to be distressing, you can contact the following support services:

Samaritans

Phone: 116 123

Website: <https://www.samaritans.org/>

Rethink Mental Illness

Website: <https://www.rethink.org/>

Aware (Ireland only)

Phone: 1800 80 48 48

Website: <https://www.aware.ie/support/>

Pieta House (Ireland only)

Phone: 1800 247 247

Website: <https://www.pieta.ie/contact/>

Shout (UK only)

Text: 85258

Mind

Website:

[https://www.mind.org.uk/](https://www.mind.org.uk/ )
