# Supplementary material for: The development and validation of the Social Attributions for Mental Illness (SAMI) scale
Source: PLoS One. 2025 May 23;20(5):e0324592. doi: 10.1371/journal.pone.0324592 (PMC12101631; doi:10.1371/journal.pone.0324592)
Supplement: S5 File — (DOCX) [file pone.0324592.s005.docx]

**Instructions for use of the Social Attributions for Mental Illness (SAMI) scale**

Participants rate the extent to which they believe each item is important as a cause for mental illness, from 1 = “not at all important as a cause” to 5 = “very important as a cause”. The SAMI has four subscales: (1) *Life circumstances* (2) *Violence/abuse* (3) *Relational challenges* and (4) *Sociopolitical turmoil*. The scale has been validated for four mental illness categories: depression, anorexia nervosa, post-traumatic stress disorder (PTSD), and schizophrenia.

**Scoring instructions**

Participant scores are calculated by averaging all items scores within a given subscale. Scores are calculated on a range of 1 to 5, with higher scores (closer to 5) indicating greater endorsement of the construct as a cause of mental illness.

*Life circumstances*: Mean score of items 1 – 13

*Violence/abuse*: Mean score of items 14 – 21

*Relational challenges*: Mean score of items 22 – 27

*Sociopolitical turmoil*: Mean score of items 28 – 33

**Sample vignettes**

*Depression is a type of mental illness. Common symptoms amongst people with depression include low mood, weight changes, difficulty sleeping, feelings of restlessness, fatigue, difficulty concentrating, and suicidal thoughts.*

*Schizophrenia is a type of mental illness. Common symptoms amongst people with schizophrenia include delusions, hallucinations, disorganised speech or behaviour, or losing the ability to do things such as going to work or school.*

*Anorexia nervosa is a type of mental illness. Common symptoms amongst people with anorexia nervosa include restricting food intake, severe weight loss, intense fear of gaining weight, and body image disturbance.*

*PTSD (posttraumatic stress disorder) is a type of mental illness. Common symptoms amongst people with PTSD include recurrent and intrusive distressing memories, recurrent distressing dreams, flashbacks, and psychological distress following specific triggers.*

*Below is a list of possible causes for PTSD.*

**Prompt**

*Below is a list of possible causes for* [depression]*.*

*Please rate each of the following items from “not at all important” to “very important” as causes for* [depression]*.*

**Full list of items**

Items should be presented in random order. Attention checks (e.g., “please select ‘3 – somewhat important’ for this item”) may be included to identify unmotivated or careless respondents, who may be screened out prior to analysis.

|  | 1 = not at all important as a cause | 2 = not very important | 3 = somewhat important | 4 = important | 5 = very important as a cause |
| --- | --- | --- | --- | --- | --- |
| **Factor 1: Life Circumstances** |  |  |  |  |  |
| 1. Being displaced from one's home |  |  |  |  |  |
| 1. Caregiving burden |  |  |  |  |  |
| 1. Inadequate social welfare supports |  |  |  |  |  |
| 1. Income inequality |  |  |  |  |  |
| 1. Insufficient leisure time, lack of time off work |  |  |  |  |  |
| 1. Lack of access to healthcare |  |  |  |  |  |
| 1. Lack of social support |  |  |  |  |  |
| 1. Poor housing conditions |  |  |  |  |  |
| 1. Poverty |  |  |  |  |  |
| 1. Reproductive struggles/ infertility |  |  |  |  |  |
| 1. Social isolation/loneliness |  |  |  |  |  |
| 1. Stressful job |  |  |  |  |  |
| 1. Unstable living conditions, moving around too often |  |  |  |  |  |
| **Factor 2: Violence/Abuse** |  |  |  |  |  |
| 1. Child emotional abuse |  |  |  |  |  |
| 1. Child neglect |  |  |  |  |  |
| 1. Child physical abuse |  |  |  |  |  |
| 1. Child sexual abuse |  |  |  |  |  |
| 1. Domestic violence |  |  |  |  |  |
| 1. Physical abuse in adulthood |  |  |  |  |  |
| 1. Sexual harassment |  |  |  |  |  |
| 1. Events that cause physical harm/injury |  |  |  |  |  |
| **Factor 3: Relational challenges** |  |  |  |  |  |
| 1. Relationship issues (with a romantic partner) |  |  |  |  |  |
| 1. Gender norms |  |  |  |  |  |
| 1. Negative influences from social groups |  |  |  |  |  |
| 1. Not fitting in |  |  |  |  |  |
| 1. Pressure and expectations |  |  |  |  |  |
| 1. Contact with other people with mental illness (in person/online) |  |  |  |  |  |
| **Factor 4: Sociopolitical Turmoil** |  |  |  |  |  |
| 1. Armed conflict |  |  |  |  |  |
| 1. Natural disasters |  |  |  |  |  |
| 1. Political instability |  |  |  |  |  |
| 1. Protests, riots, and revolutions |  |  |  |  |  |
| 1. Stress related to migration |  |  |  |  |  |
| 1. Violence within the neighbourhood |  |  |  |  |  |
