# Supplementary material for: The development and validation of the Social Attributions for Mental Illness (SAMI) scale
Source: PLoS One. 2025 May 23;20(5):e0324592. doi: 10.1371/journal.pone.0324592 (PMC12101631; doi:10.1371/journal.pone.0324592)
Supplement: S6 File — (DOCX) [file pone.0324592.s006.docx]

**Demographic information**

Table: Demographic information for sample 1 (S1, *n* = 250), sample 2 (S2, *n* = 250), and total sample (*N* = 500)

|  |  | S1 | S2 | Total |
| --- | --- | --- | --- | --- |
| Age | Range | 18 - 82 | 18 - 80 | 18 - 82 |
|  | *M* | 36.54 | 39.16 | 38.85 |
|  | *SD* | 13.93 | 13.85 | 13.89 |
| Gender | Male | 115 | 124 | 239 |
|  | Female | 130 | 119 | 249 |
|  | Other | 1 | 2 | 3 |
| Political affiliation | Left-wing | 119 | 109 | 228 |
|  | Centre | 98 | 104 | 202 |
|  | Right-wing | 31 | 35 | 66 |
| Nationality | UK | 190 | 200 | 390 |
|  | Ireland | 9 | 12 | 21 |
|  | Other | 25 | 15 | 40 |
| Ethnicity | White Irish/British | 201 | 207 | 408 |
|  | White other | 20 | 13 | 33 |
|  | Black Irish/British | 3 | 2 | 5 |
|  | Black other | 3 | 1 | 4 |
|  | Asian Irish/British | 5 | 4 | 9 |
|  | Asian other | 7 | 12 | 19 |
|  | Other/Mixed | 9 | 7 | 16 |
| Education | Primary education | 4 | 1 | 5 |
|  | Junior secondary education | 2 | 5 | 7 |
|  | Higher secondary education | 54 | 56 | 110 |
|  | Third level education | 135 | 143 | 278 |
|  | Postgraduate education | 52 | 44 | 96 |
| Understanding of depression | Had never heard of it | 0 | 1 | 1 |
|  | Knew a little | 105 | 104 | 209 |
|  | Knew a lot | 128 | 132 | 150 |
|  | Expert | 11 | 10 | 21 |
| Understanding of schizophrenia | Had never heard of it | 2 | 1 | 3 |
|  | Knew a little | 210 | 218 | 428 |
|  | Knew a lot | 33 | 24 | 57 |
|  | Expert | 3 | 1 | 4 |
| Understanding of PTSD | Had never heard of it | 1 | 1 | 2 |
|  | Knew a little | 188 | 176 | 364 |
|  | Knew a lot | 56 | 62 | 118 |
|  | Expert | 3 | 4 | 7 |
| Understanding of anorexia nervosa | Had never heard of it | 3 | 9 | 12 |
|  | Knew a little | 183 | 194 | 377 |
|  | Knew a lot | 59 | 41 | 100 |
|  | Expert | 5 | 2 | 7 |
| Knows someone with | Depression | 187 | 177 | 364 |
|  | PTSD | 65 | 66 | 74 |
|  | Anorexia nervosa | 56 | 49 | 131 |
|  | Schizophrenia | 32 | 42 | 105 |
|  | None | 48 | 60 | 108 |
| If yes, was this person | An acquaintance | 60 | 67 | 127 |
|  | A close friend | 74 | 71 | 145 |
|  | A family member | 106 | 80 | 186 |
|  | Yourself | 77 | 63 | 140 |
