# Supplementary material for: The development and validation of the Social Attributions for Mental Illness (SAMI) scale
Source: PLoS One. 2025 May 23;20(5):e0324592. doi: 10.1371/journal.pone.0324592 (PMC12101631; doi:10.1371/journal.pone.0324592)
Supplement: S7 File — (DOCX) [file pone.0324592.s007.docx]

**Validity Analysis Results**

Table 1: Convergent and divergent validity assessment

|  | | **PTSD** | | | | | | **Anorexia** | | | | | | **Depression** | | | | | | **Schizophrenia** | | | | | |
| --- | --- | --- | --- | --- | --- | --- | --- | --- | --- | --- | --- | --- | --- | --- | --- | --- | --- | --- | --- | --- | --- | --- | --- | --- | --- |
|  | SAMI | *b* | *B* | *SE* | *t* | *p* | $R^{2}$ | *b* | *B* | *SE* | *t* | *p* | $R^{2}$ | *b* | *B* | *SE* | *t* | *p* | $R^{2}$ | *b* | *B* | *SE* | *t* | *p* | $R^{2}$ |
| *MIAQ* | *S1* | **.68** | **.86** | **.03** | **26.80** | **< .001** | **.74** | .80 | .88 | .03 | 28.56 | **< .001** | .77 | **.82** | **.83** | **.03** | **23.78** | **< .001** | **.70** | **.94** | **.91** | **.03** | **34.84** | **< .001** | **.83** |
| *Social/stress* | *S2* | **.56** | **.46** | **.07** | **8.25** | **< .001** | **.22** | **.72** | **.81** | **.03** | **21.44** | **< .001** | **.65** | **.79** | **.67** | **.06** | **14.34** | **< .001** | **.45** | **.76** | **.85** | **.03** | **25.59** | **< .001** | **.73** |
|  | *S3* | **.64** | **.80** | **.03** | **21.33** | **< .001** | **.65** | **.77** | **.68** | **.05** | **14.51** | **< .001** | **.46** | **.72** | **.81** | **.03** | **21.84** | **< .001** | **.66** | **.92** | **.88** | **.03** | **29.72** | **< .001** | **.78** |
|  | *S4* | **.50** | **.55** | **.05** | **10.25** | **< .001** | **.30** | **.62** | **.77** | **.03** | **19.15** | **< .001** | **.60** | **.48** | **.65** | **.04** | **13.35** | **< .001** | **.42** | **.85** | **.85** | **.03** | **25.43** | **< .001** | **.72** |
| *MIAQ* | *S1* | **.78** | **.67** | **.05** | **14.36** | **< .001** | **.45** | **.75** | **.69** | **.05** | **14.86** | **< .001** | **.47** | **.67** | **.52** | **.07** | **9.51** | **< .001** | **.27** | **.21** | **.31** | **.04** | **5.07** | **< .001** | **.09** |
| *Heredity/* | *S2* | **.44** | **.25** | **.11** | **4.08** | **< .001** | **.06** | **.59** | **.55** | **.06** | **1.29** | **< .001** | **.30** | **.71** | **.46** | **.09** | **8.23** | **< .001** | **.21** | **.16** | **.27** | **.04** | **4.40** | **< .001** | **.07** |
| *Biological* | *S3* | **.78** | **.68** | **.05** | **14.43** | **< .001** | **.46** | **.59** | **.43** | **.08** | **7.51** | **< .001** | **.19** | **.62** | **.54** | **.06** | **9.98** | **< .001** | **.29** | **.19** | **.27** | **.04** | **4.48** | **< .001** | **.08** |
|  | *S4* | **.44** | **.32** | **.08** | **5.37** | **< .001** | **.10** | **.64** | **.66** | **.05** | **13.86** | **< .001** | **.44** | **.52** | **.54** | **.05** | **1.01** | **< .001** | **.29** | **.18** | **.26** | **.04** | **4.32** | **< .001** | **.07** |

Where b = unstandardised coefficient, B = standardised coefficient, SE = standard error, S1 = SAMI (1) *life circumstances*, S2 = SAMI (2) *violence/abuse,* S3 = SAMI (3) *relational challenges*, S4 = SAMI (4) *sociopolitical turmoil.* The analysis was preformed using sample 2 data.

Table 2: Discriminant validity assessment

|  | **PTSD** | | | | | | **Anorexia** | | | | | | **Depression** | | | | | | **Schizophrenia** | | | | | |
| --- | --- | --- | --- | --- | --- | --- | --- | --- | --- | --- | --- | --- | --- | --- | --- | --- | --- | --- | --- | --- | --- | --- | --- | --- |
|  | *M1* | *M2* | *t* | *p* | 95% CI | d | *M1* | *M2* | *t* | *p* | 95% CI | d | *M1* | *M2* | *t* | *p* | 95% CI | d | *M1* | *M2* | *t* | *p* | 95% CI | d |
| ***S1*** | 2.71 | 2.56 | .79 | .215 | -.17 | .15 | **2.69** | **2.24** | **2.26** | **.012** | **1.55** | **.44** | **3.98** | **3.61** | **2.94** | **.002** | **.16** | **.57** | **3.98** | **3.61** | **2.94** | **.002** | **.16** | **.22** |
| ***S2*** | **4.50** | **4.24** | **2.00** | **.024** | **.04** | **.39** | **3.80** | **3.37** | **2.11** | **.018** | **.09** | **.41** | **4.60** | **4.37** | **2.29** | **.012** | **.06** | **.44** | **4.60** | **4.37** | **2.29** | **.012** | **.06** | **-.01** |
| ***S3*** | 2.43 | 2.40 | .14 | .446 | -.30 | .03 | **3.72** | **3.18** | **3.45** | **<.001** | **.28** | **.67** | **3.55** | **3.24** | **2.32** | **.011** | **.09** | **.45** | **3.55** | **3.24** | **2.32** | **.011** | **.09** | **.11** |
| ***S4*** | **3.73** | **3.08** | **4.10** | **<.001** | **.39** | **.80** | 2.09 | 1.75 | 1.50 | .07 | -.04 | .29 | **3.51** | **2.87** | **4.03** | **<.001** | **.38** | **.78** | **3.51** | **2.87** | **4.03** | **<.001** | **.38** | **.35** |

Where IV = political affiliation, M1 = sample 1 mean (left-wing), M2 = sample 2 mean (right-wing), d = Cohen’s d, CI = confidence interval, S1 = SAMI (1) *life circumstances*, S2 = SAMI (2) *violence/abuse,* S3 = SAMI (3) *relational challenges*, S4 = SAMI (4) *sociopolitical turmoil.* The analysis was preformed using sample 2 data.

**Reliability analysis results**

Table 3: Internal consistency assessment and descriptive statistics for each SAMI subscale

| **SAMI Subscale** | **Cronbach’s alpha** | ***M*** | ***SD*** |
| --- | --- | --- | --- |
| *Life circumstances* | 0.96 | 3.00 | 1.08 |
| *Violence/abuse* | 0.95 | 4.04 | 1.02 |
| *Relational challenges* | 0.89 | 3.00 | 1.05 |
| *Sociopolitical turmoil* | 0.92 | 2.82 | 1.17 |

The analysis was preformed using sample 2 data.
